# The stroke mothership model survived during COVID-19 era: an observational single-center study in Emilia-Romagna, Italy

**DOI:** 10.1007/s10072-020-04754-2

**Published:** 2020-10-08

**Authors:** Andrea Zini, Michele Romoli, Mauro Gentile, Ludovica Migliaccio, Cosimo Picoco, Oscar Dell’Arciprete, Luigi Simonetti, Federica Naldi, Laura Piccolo, Giovanni Gordini, Francesco Tagliatela, Vincenzo Bua, Luigi Cirillo, Ciro Princiotta, Carlo Coniglio, Carlo Descovich, Pietro Cortelli

**Affiliations:** 1 IRCCS Istituto delle Scienze Neurologiche di Bologna, Department of Neurology and Metropolitan Stroke Center, “C.A. Pizzardi” Maggiore Hospital, Largo Nigrisoli 2, 40133 Bologna, Italy; 2 Neurology Clinic, University of Perugia, Perugia, Italy; 3 Department of Anaesthesia, Intensive Care and Emergency Medical Services, Maggiore Hospital, Bologna, Italy; 4 Neuroradiology Unit, Maggiore Hospital, IRCCS Istituto delle Scienze Neurologiche di Bologna, Bologna, Italy; 5 Neuroradiology Unit, Bellaria Hospital, IRCCS Istituto delle Scienze Neurologiche di Bologna, Bologna, Italy; 6 DIMES, Department of Specialty, Diagnostic and Experimental Medicine, University of Bologna, Bologna, Italy; 7 Department of Clinical Governance and Quality, Bologna Local Healthcare Authority, Bologna, Italy; 8 Department of Biomedical and Neuromotor Sciences, Alma Mater Studiorum-University of Bologna, Bologna, Italy; 9 IRCCS Istituto delle Scienze Neurologiche di Bologna, Bologna, Italy

**Keywords:** Ischemic stroke, Transient ischemic-attack, Epidemiology, COVID-19

## Abstract

**Introduction:**

A reduction of the hospitalization and reperfusion treatments was reported during COVID-19 pandemic. However, high variability in results emerged, potentially due to logistic paradigms adopted. Here, we analyze stroke code admissions, hospitalizations, and stroke belt performance for ischemic stroke patients in the metropolitan Bologna region, comparing temporal trends between 2019 and 2020 to define the impact of COVID-19 on the stroke network.

**Methods:**

This retrospective observational study included all people admitted at the Bologna Metropolitan Stroke Center in timeframes 1 March 2019–30 April 2019 (cohort-2019) and 1 March 2020–30 April 2020 (cohort-2020). Diagnosis, treatment strategy, and timing were compared between the two cohorts to define temporal trends.

**Results:**

Overall, 283 patients were admitted to the Stroke Center, with no differences in demographic factors between cohort-2019 and cohort-2020. In cohort-2020, transient ischemic attack (TIA) was significantly less prevalent than 2019 (6.9% vs 14.4%, *p* = .04). Among 216 ischemic stroke patients, moderate-to-severe stroke was more represented in cohort-2020 (17.8% vs 6.2%, *p* = .027). Similar proportions of patients underwent reperfusion (45.9% in 2019 vs 53.4% in 2020), although a slight increase in combined treatment was detected (14.4% vs 25.4%, *p* = .05). Door-to-scan timing was significantly prolonged in 2020 compared with 2019 (28.4 ± 12.6 vs 36.7 ± 14.6, *p* = .03), although overall timing from stroke to treatment was preserved.

**Conclusion:**

During COVID-19 pandemic, TIA and minor stroke consistently reduced compared to the same timeframe in 2019. Longer stroke-to-call and door-to-scan times, attributable to change in citizen behavior and screening at hospital arrival, did not impact on stroke-to-treatment time. Mothership model might have minimized the effects of the pandemic on the stroke care organization.

## Background

The COVID-19 pandemic has forced a comprehensive reorganization of stroke networks to provide optimal care while containing the risk of transmission. COVID-free and COVID-positive pathways have been developed, with potential impact on management of time-dependent diseases [1, 2]. Increasing reports are available to date on the variations in stroke management throughout the pandemic, with results suggesting a potential gross reduction of the incidence as well as of the ratio of eligible patients, potentially attributable to the lengthening of rescue belt [3–7]. A preliminary report from Shanghai, China, demonstrated that thrombectomies were halved during the first month of the pandemic [1]. However, the interpretation of results from following investigations has been limited by study design, with some counting scanned patients[3], and some providing no specific methods to support findings [4, 5].

Italy has been one of the most severely hit countries. Severe restrictions, including logistic limitations and physical distancing, might have impacted on personal belief on the need for in-person consultation, as well as on the performance of the stroke belt (personnel and facilities involved in the stroke time-dependent management). Our region, Emilia-Romagna, is one of the most affected, with more than 27,000 cases and 4000 deaths.

Here, we provide a direct comparison for stroke-code admissions, hospitalizations, and timing of rescue/treatment for ischemic stroke patients in the Bologna Metropolitan Stroke Network, comparing temporal trends between 2019 and 2020 to define differences in stroke treatment, time-dependent pathway management, and overall stroke-belt performance during the COVID-19 pandemic.

## Patients and methods

### Design and setting

This retrospective electronic-record-based study included all patients admitted with a stroke code at the emergency department (ED) of the Bologna Metropolitan Stroke Center, Ospedale Maggiore, between 1 March 2019 and 30 April 2019 and 1 March 2020 and 30 April 2020. The study was approved by the Local Ethics Committee, with patient consent waived given to the retrospective anonymous collection of data, according to Italian regulations. Since 2018, the Metropolitan Stroke Network was based on a direct mothership paradigm [8], to serve a population of approximately 1 million inhabitants. Twelve hospitals were reorganized, and one Comprehensive Stroke Center was established at the Maggiore Hospital. The Comprehensive Stroke Center was programmed to provide reperfusion treatments, with 5 stroke units in spoke hospitals networked with the Hub to receive and manage treated and untreated patients. The Stroke Network has been functioning since early 2018, with no changes in direct mothership model ever since. Out-of-hospital rescue services can count on ambulance services and helicopter in cases of expected transport time exceeding 45 min. In-hospital assessment is performed at arrival by the team of neurologists and dedicated stroke physicians. Advanced imaging is available 365/24/7, with brain computerized tomography (CT) scan followed by CT perfusion (CTP) and CT angiogram (CTA), with tissue-based windows implemented to help in defining treatment indication when needed. During the COVID-19 pandemic, the Emergency Department (ED) planned separated pathways for COVID-19-suspected vs COVID-19-negative patients, with dedicated in-hospital diagnostic facilities. COVID-19 screening included rapid nasopharyngeal swab and body temperature check. High-resolution lung CT scan was performed to all suspected patients as well as to those undergoing reperfusion strategies, in order to orient the patient towards the appropriate angio-suite in case of endovascular intervention. There was no change in brain imaging/anesthetic protocol, staff, or seniority of staff during the pandemic.

### Patient identification and temporal trends considered

The March–April timeframe was selected to allow direct comparison between a period of standard workload (March–April 2019, cohort-2019) and the workload during the COVID-19 pandemic, after logistic restrictions were put into place by national and local government (March–April 2020, cohort-2020). We aimed to evaluate the performance of the stroke belt, and therefore programmed the extraction of all people admitted via stroke code during the timeframe considered. Therefore, data were derived from the electronic regional health system with anonymous record linkage, for which the Internal Ethical Committee waived the need for patient informed consent. Once obtained all stroke codes activated via 118/ED call, we cross-linked data with hospital records, reviewed charts and ICD9 diagnosis, in order to extract the pool of patients suffering from an ischemic stroke, independently from treatment received. ICD9 coding for stroke and reperfusion strategies at admission and discharge were matched to define treatments provided, refine diagnosis, and avoid inclusion of mimics [9]. As a further step to confirm the robustness of the cohort, we cross-checked patients receiving treatment with records of consecutively enrolled patients in SITS database (sitsinternational.org/registries/sits-thrombolysis/, sitsinternational.org/registries/sits-thrombectomy/) and the Italian Registry of Endovascular Treatment in Acute Stroke (IRETAS).

Data regarding date of stroke, rescue belt (personnel and facilities involved in territorial emergency services), arrival in hospital, baseline National Institute of Health Stroke Scale score (NIHSS), stroke severity (minor 0–5; moderate 6–15; moderate to severe 16–20; severe > 20), diagnostics, timing of treatment, and type of treatment (intravenous thrombolysis (IVT), endovascular thrombectomy (EVT), or both IVT + EVT) were collected and compared between the two cohorts selected.

Statistical analysis was performed with SPSS and R software. Descriptive statistics are presented for continuous variables as means and standard deviations, and were tested for normal distribution. Categorical variables are presented as counts and percentages. *χ*^2^ and Student’s *T* test were used for univariate inference as appropriate, with Bonferroni adjustment. Significance level was set to be 0.05.

## Results

ED admissions were 10,689 in March–April 2019 and 6897 in March–April 2020. During the 4 months considered, 283 patients (*n*_2019_ = 138, *n*_2020_ = 145) were admitted to the Comprehensive Stroke Center with a suspected diagnosis of stroke. No differences in NIHSS, age, and gender distribution emerged between cohorts (Table 1).

**Table 1:** Characteristics of included cohorts (whole population)

	Cohort-2019 (*n* = 138)	Cohort-2020 (*n* = 145)
Female, *n* (%)	68 (49.3%)	67 (46.2%)
Age, mean ± SD	75.2 ± 13.8	71.7 ± 18
Cerebrovascular disease		
Intracerebral hemorrhage, *n* (%)	21 (15.2%)	17 (11.7%)
Transient ischemic attack, *n* (%)	20 (14.5%)	10 (6.9%)*
Ischemic stroke, *n* (%)	97 (70.3%)	118 (81.4%)*
NIHSS, mean ± SD	7.6 ± 7.1	9.2 ± 7.6
Month		
March, *n* (%)	62 (44.9%)	72 (49.7%)
April, *n* (%)	76 (55.1%)	73 (50.3%)

*p < 0.05

*NIHSS* National Institute of Health Stroke Scale

TIA admitted to the ED were 65 in March–April 2019 and 38 in March–April 2020 (relative variation = − 41.5%). Consequently, TIA admitted to the Stroke Unit were overall under-represented (6.9% vs 14.5%, *p* = .04), despite similar rates of hospitalization for high risk TIA (30.7% vs 26.3%, *p* = .9). Intracerebral hemorrhage remained grossly unchanged (Table 1).

**Fig. 1:**
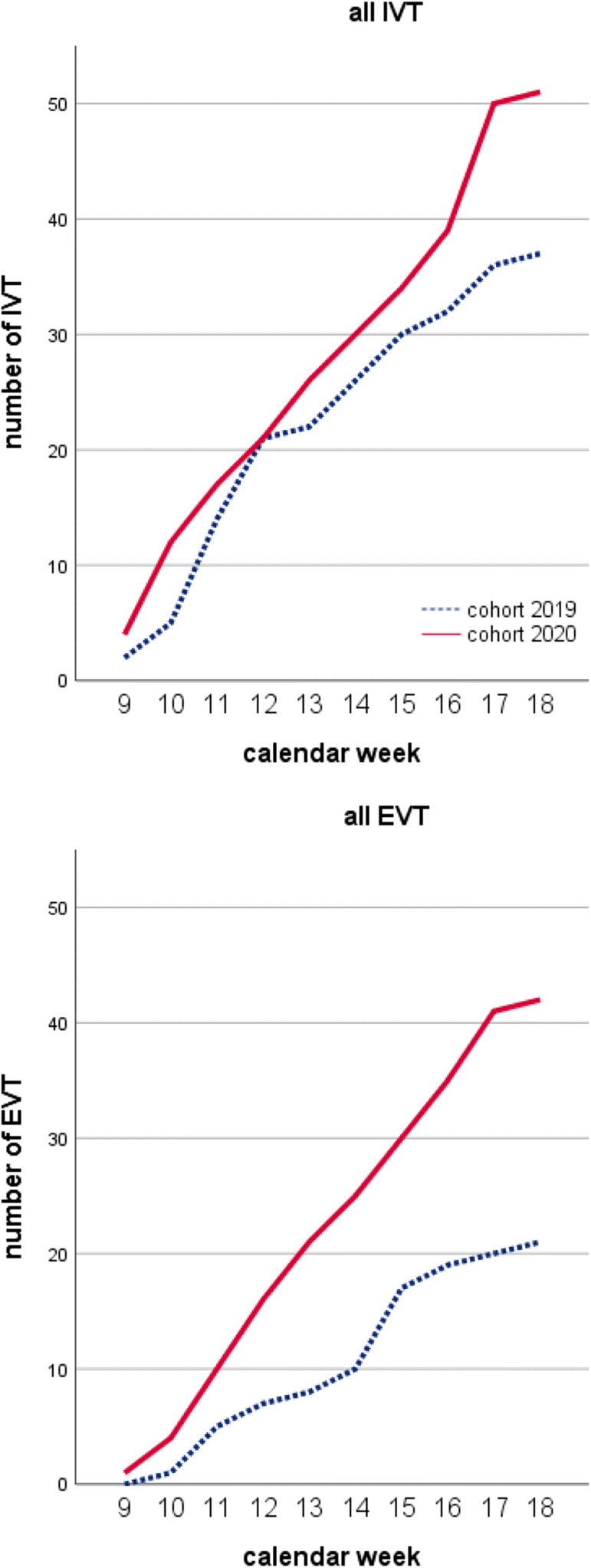
Temporal trends of reperfusion treatments across weeks in 2019 and 2020 cohorts, starting from 1/3 (week 9) to 30/4 (week 18). EVT endovascular thrombectomy, IVT intravenous thrombolysis

Overall, 215 patients were diagnosed with ischemic stroke, 97 in cohort-2019 and 118 in cohort-2020, with higher prevalence of patients with moderate to severe stroke in cohort-2020 (17.8% vs 6.2%, *p* = .027; Table 2). Similar proportions of patients underwent reperfusion (2019 = 45.4% vs 2020 = 53.4%), with significant increase in combined treatment (14.4% vs 25.4%, *p* = .05) and overall EVT (21.6% vs 35.6%, *p* = .025, Fig. 1). Stroke-to-call time was marginally longer in cohort-2020 (65.5 ± 104.3 min vs 33.7 ± 40.2 min, *p* = .06), with higher variability in 2020. Timing of treatment and rescue did not differ significantly between the cohorts. On the contrary, door-to-scan timing was significantly prolonged in 2020 compared with 2019 (28.4 vs 36.7 min, *p* = .03). However, stroke-to-recanalization remained unchanged.

**Table 2:** Demographic and clinical details, treatment and timings and timings for patients admitted with acute ischemic stroke

	Cohort-2019 (*n* = 97)	Cohort-2020 (*n* = 118)
Female, *n* (%)	54 (55.1%)	56 (47.5%)
Age, mean ± SD	76.6 ± 13.7	72.9 ± 16.6
NIHSS, mean ± SD	8.9 ± 7.2	9.5 ± 7.4
Stroke severity, *n* (%)		
Minor stroke	2 (2.1%)	0 (0%)
Moderate stroke	78 (80.4%)	88 (74.6%)
Moderate to severe	6 (6.2%)	21 (17.8%)*
Severe	11 (11.3%)	9 (7.6%)
Reperfusion, *n* (%)	44 (45.4%)	63 (53.4%)
IVT only	23 (23.7%)	21 (17.8%)
EVT only	7 (7.2%)	12 (10.2%)
IVT + EVT	14 (14.4%)	30 (25.4%)*
All IVT	37 (38.1%)	51 (43.2%)
All EVT	21 (21.6%)	42 (35.6%)*
Reperfusion timing, mean ± SD^#^		
Stroke to call	33.7 ± 40.2	65.5 ± 104.3
Call to rescue	15.4 ± 7.9	12.9 ± 5.8
Rescue to door	53.2 ± 38	44 ± 26.6
Door to scan	28 ± 12.6	36.7 ± 14.6*
Door to needle	67.8 ± 20.8	72.6 ± 34.3
Stroke to needle	164.5 ± 51.8	173.9 ± 71.2
Door to groin	116.9 ± 39	118.8 ± 55.7
Groin to recanalization	53.1 ± 35.6	66.8 ± 43.9
Door to recanalization	170 ± 53.4	186 ± 82.8
Stroke to recanalization	337.7 ± 310.2	361 ± 270

*p value < 0.05

^#^Complete timing data available for 33/44 patients in cohort-2019 and 49/63 patients in cohort-2020

## Discussion

This observational before−after study comparing access to reperfusion strategies and treatment in a single Comprehensive Stroke Center demonstrates that the performance of the mothership-based stroke network was preserved during the pandemic.

First, we observed a consistent reduction in number of TIA and minor stroke admitted to the emergency department. Policies minimizing non-essential in-person provider–patient interactions might have impacted, as well as personal reticence to access the ED during the pandemic. If so, a consistent increase in patients searching medical attention weeks of months after TIAs or minor stroke might be predictable once the pandemic is over. For that time, our stroke care network has to meet the need of appropriate secondary prevention and rehabilitation.

Second, our results support the elasticity of a mothership paradigm, which maintained its performance over the pandemic. This is in contrast with previous studies reporting reduction in hospitalized patients and revascularization treatments [3–7], which however were based on different logistic paradigms. Although delay in emergency calls was observed, possibly reflecting a change in behavior of the population, the performance of the stroke belt was substantially preserved. The marginally longer stroke-to-call time depended on larger variability in the cohort-2020, suggesting that stroke awareness campaign might be implemented during the pandemic to reinforce a timely access to emergency services. Citizens’ stroke awareness is paramount to guarantee access to treatment, and recent regional campaigns in 2014 [10] and in 2018 (https://salute.regione.emilia-romagna.it/campagne/ictus-vedo-riconosco-chiamo) might have contributed to early recognition, and encouraged calling emergency services.

The in-hospital path was characterized by longer door-to-scan time, which might be plausibly attributable to the need of a pre-screening station addressing body temperature and stratification for COVID-19. However, delay in scanning was minimal, and did not impact on stroke-to-treatment timing, which was similar to 2019. To this extent, the use of CT scan might ease and shorten the path compared with brain MRI during the pandemic.

Regarding reperfusion strategies, a significant increase in EVT was found in cohort-2020 compared with cohort-2019. Several factors might have contributed: (i) the progressive improvement of a recently developed stroke network, (ii) an improvement in skills for endovascular approach in cases of distal occlusions, (iii) an increase in stroke severity observed in cohort-2020, with potential higher rates of large vessel occlusion. The global stroke-to-treatment time was similar in 2019 vs 2020 and it might reflect the adherence to the predefined stroke network also during the COVID-19 outbreak. Overall, the boat seemed to have survived the COVID-19 storm, although in-hospital path can still be improved/shortened [11].

Limitations to this study can be found in the small sample size and in the electronic-derived cohorts, which might have to some extent underestimated the prevalence of stroke. However, the paradigm seemed consistent with prospective data, since no patient was excluded according to ICD9 coding, and similar retrieve strategies were applied to both cohorts. Second, the lack of analysis of neuroimaging data limits the interpretation of the increase in EVT treatment approach, which however seems marginally significant and might well reflect the evolution of endovascular procedures towards more distal occlusions over time.

## Conclusion

COVID-19 pandemic has represented and still seems a bad bargain to get the best out of. The management of stroke should be organized to comply with the time-dependent nature of the disease. Resources should be allocated, and mothership or drip-and-ship paradigms should be critically appraised to guarantee optimal care and limit delays due to pandemic circumstances.

## Data Availability

Requests for data sharing can be directed to the corresponding author
